# You “R/R” What You Eat: Effects of High‐Fiber, Low‐Starch Diet Change on Regurgitation and Reingestion and Coprophagy in Zoo‐Housed Gorillas

**DOI:** 10.1002/zoo.21885

**Published:** 2024-12-19

**Authors:** Lisa P. Barrett, Jennifer D'Agostino, Heather Guillory, Kimberly Leser, Laura Bottaro, Rebecca J. Snyder

**Affiliations:** ^1^ Oklahoma City Zoo and Botanical Garden Oklahoma City Oklahoma USA

**Keywords:** animal welfare, coprophagy, *Gorilla gorilla gorilla*, remastication, zoo

## Abstract

Regurgitation and reingestion (R/R) and coprophagy are common behaviors exhibited by primates living in human care. To reduce this undesirable behavior in two troops of western lowland gorillas (*Gorilla gorilla gorilla*), the diet was modified by increasing fiber (by increasing browse) and decreasing starch (by reducing but not eliminating biscuits). We monitored behavior before, 3 weeks after, and 1 year after the diet change. One year after the diet change, the family group's diet was modified by adding more fruit to facilitate training. We documented significantly increased feeding activity for both troops, with time spent feeding doubling for one troop. Some individuals initially increased R/R or coprophagy behaviors but these increases were not significant, and 1 year after the diet change R/R was significantly reduced in the silverback male who had been known to exhibit R/R up to multiple times per day. In the family troop, coprophagy later decreased when animals were reunited and spent more time outdoors (for reasons unrelated to the diet change study), but this was not significant. This is the first published study to assess the behavioral effects of a high‐fiber, low‐starch diet on gorillas one full year after the diet change was made, and we demonstrated that the diet continued to positively impact some individuals' behavior. We discuss implications of our findings and suggest future directions for institutions that seek to reduce these behaviors in primates without necessarily completely eliminating biscuits from their diets.

## Summary

- We observed behavior before and after increasing fiber and reducing starch in diets of Western lowland gorillas in human care.
- After the diet change, gorillas spent more time feeding and we saw effects on regurgitation and coprophagy.
- Our observations highlight the importance of long‐term observations and the important effects of moderate changes in diet.

## Graphical abstract

In this study, we increased fiber (by increasing browse) and decreased starch (by reducing but not eliminating biscuits) to reduce regurgitation and reingestion and coprophagy. We monitored behavior before, 3 weeks after, and 1 year after the diet change and found important effects on behavior.

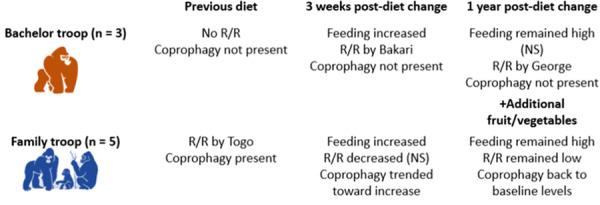

## 1 Introduction

Regurgitation and reingestion (R/R) (Video S1) is a well‐documented, undesirable behavior of apes and other primates living in human care. R/R has been documented in several nonhuman primate species, including all of the great ape species, with an estimated 60%–65% of gorillas in AZA facilities exhibiting the behavior (reviewed in Tennant et al. 2021). R/R has never been observed in wild gorillas (Hill 2018; Lukas 1999), but it has been documented in the wild in the proboscis monkey (*Nasalis larvatus*, Matsuda et al. 2011) and the indri lemur (*Indri indri*, Randrianarison et al. 2022). While there are no proven detrimental health effects from R/R in gorillas, there are numerous deleterious health effects noted in humans who exhibit a similar behavior as part of rumination syndrome (reviewed in Lukas 1999; Hill 2018). Detrimental health effects of rumination syndrome in humans include dental erosion, esophageal strictures, ulcers and pulmonary aspiration. Many of these health effects are caused by regurgitation of partially digested food material containing stomach acid. Hill (2009) demonstrated that stomach acid is present in the regurgitated food material in gorillas, so the potential for long‐term health effects is serious. In addition to potential health effects of R/R, it is thought to evoke negative feelings for zoo visitors (Akers and Schildkraut 1985). Moreover, R/R is often studied in conjunction with coprophagy behavior, which is a naturally occurring behavior in wild apes but occurs more often in human care than in the wild and is objectionable for visitors at zoos (reviewed in Hill 2018). R/R and coprophagy are thus considered undesirable behaviors by many practitioners.

No specific cause has been linked to R/R behavior but it is largely believed that nutrition, including nutritional content and presentation of diet, is a primary factor (e.g., Tennant et al. 2021; Less et al. 2014a, 2014b). The diet of wild gorillas is high in fiber and low in starch consisting primarily of leaves, bark, stems, and seeds (Smith, Remis, and Dierenfeld 2014; Less et al. 2014a). Conversely, the diet of gorillas in human care is relatively low in fiber (Popovich and Dierenfeld 1997) and high in simple carbohydrates (sugars and starches) (Less et al. 2014a, 2014b) and may include items that are not part of a gorilla's natural diet (e.g., milk, yogurt). Additionally, gorillas in human care are typically fed multiple discrete meals throughout the day rather than a continuous supply of food (AZA Gorilla Species Survival Plan 2017). Consequently, gorillas in human care spend a significantly lower proportion of the day foraging and a larger proportion of the day inactive (Less et al. 2014a; Masi, Cipolletta and Robbins 2009; Magliocca and Gautier‐Hion 2002). The combination of a diet lower in fiber (which also contains items higher in starch) and relative inactivity as a result of less time spent feeding/foraging, has been identified as a possible factor in instigating R/R behavior (Lukas 1999; Struck et al. 2007). Fortunately, diet manipulation studies have shown a significant decrease or complete elimination in R/R behavior with exclusion of certain items from the diet such as milk, fruit, and commercial biscuits (e.g., Mulder et al. 2016; Less et al. 2014a, 2014b; Lukas et al. 1999), yet recent research discovered that two‐thirds of non‐infant, zoo‐housed gorillas still exhibit R/R, highlighting the need for more work to eliminate R/R (Tennant et al. 2021).

Less et al. (2014a) proposed that a decrease in R/R could lead to an increase in coprophagy if there is more stool produced with the diet change and/or if the diet change is perceived as a period of food scarcity. It is also possible that coprophagy, which is more commonly seen during wet/rainy days when gorillas are potentially less active, fulfills a lack of foraging opportunities (Akers and Schildkraut 1985). Thus, we predicted that we would see an increase in foraging activity and therefore less coprophagy.

At the Oklahoma City Zoo, the 30‐year‐old male silverback in the western lowland gorilla (*Gorilla gorilla gorilla*) family troop (Togo) historically exhibited high levels of R/R behavior (i.e., up to several times per day). The bachelor troop of three male silverback western lowland gorillas (Bakari, Boenje, and George) occasionally exhibited R/R (i.e., 2–3 times per month), as well. Additionally, the family troop exhibited coprophagy daily and most members of the troop performed it (all members except Togo and Ndjole). The bachelor troop exhibited coprophagy 1–2 times per week. Due to the undesirable nature of these behaviors from a visitor perspective as well as possible detrimental health effects from chronic R/R, we sought to eliminate R/R and coprophagy by manipulating the diet of our gorilla troops (following Less et al. 2014a, 2014b; Wiard 1992). We expected that modifying the diet of the silverback gorilla to reduce fruit, yogurt, starchy vegetables, and commercial biscuits, and increase fiber by adding additional browse and alfalfa hay would significantly reduce or eliminate regurgitation and reingestion behavior and coprophagy behavior. We also predicted that both troops would increase their time spent feeding as a result of spending more time processing increased browse in their diets.

## 2 Materials and Methods

### 2.1 Subjects

Subjects were two troops of western lowland gorillas at the Oklahoma City Zoo, Oklahoma City, OK, USA: (1) a bachelor group (*n* = 3) and (2) a family group (*n* = 5) (Table 1). Both troops were housed in indoor and outdoor enclosures. Both troops sometimes had access to both indoor and outdoor enclosures, and both troops sometimes had access to just an indoor or an outdoor enclosure. Indoor enclosures were large rooms in which the gorillas were viewable to zoo guests. The rooms were 38 and 40 square meters in size, surrounded by cement block walls on four sides, glass walls on two sides, and concrete floors. They contained climbing structures, hammocks and drinking water sources. Animal doors led from these rooms to outdoor enclosures. For the family troop, the outdoor enclosure was 2546 square meters in size. The bachelor troop's outdoor enclosure was 915 square meters. Both outdoor enclosures were surrounded by dry moats, had grass and dirt substrates, large concrete fabricated boulders, deadfall, and varied topography. The family troop enclosure had several tall trees growing in it. The bachelor troop outdoor enclosure contained a large climbing structure with a hammock. The gorillas were fed on habitat each morning with a scatter feed.

**Table 1:** Subject information. All subjects were born in human care.

Bachelor troop	Sex	Age at midpoint of study (June 2020)	Hours observed
George	Male	16	120
Bouendje	Male	13	120
Bakari	Male	14	120
**Family troop**			
Togo	Male	32	120
Ndjole	Female	25	120
Mikella	Female	16	120
Rubi	Female	6	120
Azinza	Female	4	120

### 2.2 Data Collection

**Figure 1:**
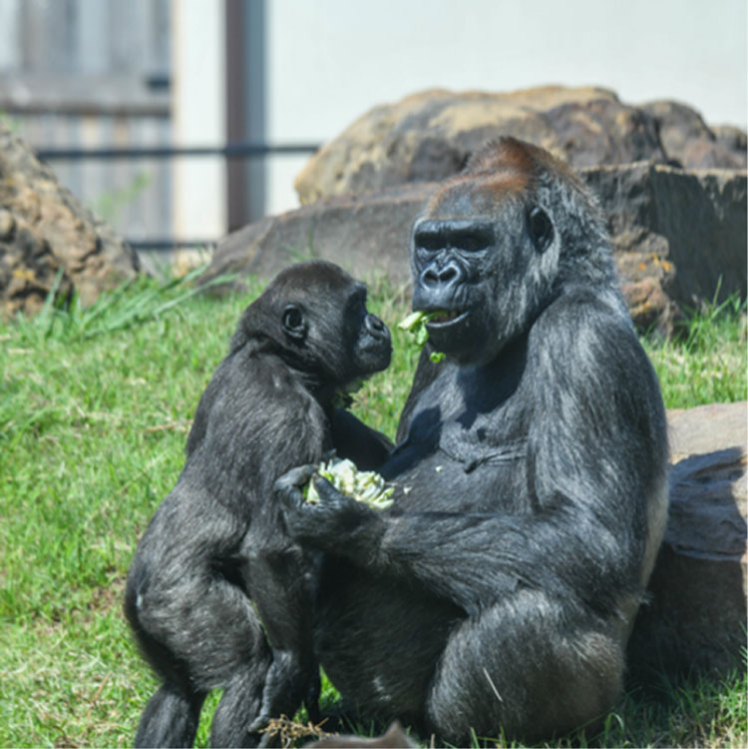
Members of family troop foraging at the Oklahoma City Zoo (Photo by Sabrina Heise/Oklahoma City Zoo and Botanical Garden).

Five observers conducted behavioral observations of all members of the family and bachelor troops (family troop foraging in Figure 1) before and 3 weeks after a diet manipulation, as well as 1 year after the diet change. For the family troop, we conducted prediet change observations June–July 2019, postdiet change observations August–October 2019, and 1‐year follow up observations August–September 2020 (when some fruit and vegetables were also added back into the diet). In 2019 during observation months, two gorillas (surrogate mother and son; excluded from the current study) were physically (but not visually or auditorily) separated from the rest of the troop because the surrogate mother was recovering from hernia repair surgery. For the bachelor troop, we conducted pre‐diet change observations June‐July 2020, post‐diet change observations August–September 2020, and 1‐year follow up observations August–October 2021. By collecting data at the same time of year across years, we aimed to reduce any potential confounds of season/temperature, and subjects did not experience any differences in fresh browse/hay availability across conditions.

Following Less et al. (2014a, 2014b) we conducted instantaneous scan sampling at 3‐min intervals during 30‐min observation sessions to record behavior and mobility (Table 2). All occurrences of R/R and coprophagy were also recorded for each animal. Please see Supplemental Information for a video of R/R behavior (Video S1). We collected 40 h of behavioral data before the start of the diet manipulation, 3 weeks after the diet change, and 1 year after the diet change (120 total hours per gorilla Table 1). Observation sessions were balanced across the following time periods: 0900–1100, 1100–1300, 1300–1500, and 1500–1700. Before the start of data collection, each observer was reliability tested with the last author until achieving an index of concordance of 85% or higher (Martin and Bateson 1993). All observations were conducted from the public viewing areas.

**Table 2:** Ethogram used during behavioral observations of the family and bachelor gorilla troops (based on Less et al. 2014a).

Behavior	Recording	Definition	Notes
Inactive	Instantaneous	Gorilla is not engaged in any active behavior, may be resting or stationary alert, includes waiting/watching at door leading from dayroom into keeper space.	
Locomote	Instantaneous	Gorilla is walking, running, or climbing. Must move at least one body length. If a gorilla is carrying another gorilla, score locomote for the gorilla doing the carrying and other for the gorilla being carried. For example, if Mikella is carrying Azinza, this is scored as locomote for Mikella and Other for Azinza.	Feed/forage takes precedence.
Feed/forage	Instantaneous	Gorilla is eating, carrying, searching for, or processing food, includes drinking.	Takes precedence over locomote.
Object manipulation	Instantaneous	Gorilla is handling an object that is not food.	If hand is resting on object, but not manipulating it, score as Inactive.
Self‐directed	Instantaneous	Gorilla is self‐grooming, scratching, licking, inspecting hair, includes plucking and consuming hair (often displayed by Bo) and holding one or both ears (often displayed by Bakari).	
Regurge and reingest	Instantaneous and All‐occurrence	Voluntary retrograde movement of food or fluid from the esophagus or stomach into the mouth or hand. One bout consists of pre‐RR behavior (hand‐shaking, stomach drumming, etc.), regurgitation of food, and then consumption of that regurgitated mass.	One bout must end and at least 5 s pass before another bout is scored.
Coprophagy	Instantaneous and All‐occurrence	Gorilla ingests feces, includes picking food out of feces and consuming it. One bout is transfer to mouth and consumption.	One bout must end and at least 5 s pass before another bout is scored.
Affiliative	Instantaneous	Gorilla is initiating or receiving allogrooming, social play, or sexual behavior, includes brief touch, pat, grasp, etc. that is not aggressive.	Takes precedence over locomote.
Agonistic	Instantaneous	Gorilla is initiating or receiving charging, chasing, chest‐beating, ground/object slapping, throwing objects, striking, includes quadrupedal tight‐lipped display posture if it appears directed at another gorilla. At least one of the animals involved is tight‐lipped.	Takes precedence over locomote.
Displace	Instantaneous	Gorilla initiates or receives approach which results in other gorilla moving away.	Takes precedence over locomote.
Other	Instantaneous	Any behavior not defined above.	
Not visible	Instantaneous	Gorilla's behavior is not visible to the observer.	
**Mobility**			
Mobile	Instantaneous	Gorilla is in the process of moving more than one body length.	
Immobile	Instantaneous	Gorilla is moving less than one body length. If Azinza or Fin is being carried, she/he is scored as immobile.	
Not visible	Instantaneous	Gorilla's behavior is not visible to the observer.	

### 2.3 Diet Change

Veterinary and primate staff implemented a high‐fiber, low‐starch diet (Table 3; Please see Supplemental Information 1 for more details). Staff weighed animals weekly to monitor closely for any weight loss associated with the diet change. Importantly, we retained some biscuits in the diet for their vitamin and mineral content—to ensure that we would not inadvertently create a nutritional deficiency if we removed them completely. In 2020, staff decided to increase some fruits and vegetables used for positive reinforcement training in the family troop (Table 3).

**Table 3:** Summary of changes in diet composition. Percentages are of total diets “as fed” by weight. Browse included elm, mulberry, or hackberry. Forage included Wild Herbivore pellet, low‐sugar cereal (i.e., cheerios or chex), air‐popped popcorn, and mixed nuts.

	Prediet change	Postdiet change	Postdiet change (1 year)
**Family Troop**			
	**Jun.–Jul. 2019**	**Aug.–Oct. 2019**	**Aug.–Sep. 2020**
Fruit	6.00%	2.80%	4.10%
Veggie	34.40%	23.00%	26.40%
Greens	34.40%	39.30%	39.60%
Biscuit	6.20%	4.60%	4.50%
Forage	1.70%	1.50%	1.20%
Browse	17.20%	28.80%	24.20%
**Bachelor troop**			
	**Jun.–Jul. 2020**	**Aug.–Sep. 2020**	**Aug.–Oct. 2021**
Fruit	5.60%	4.40%	4.40%
Veggie	33.50%	26.30%	26.30%
Greens	36.90%	39.50%	39.50%
Biscuit	5.60%	4.40%	4.40%
Forage	1.70%	1.30%	1.30%
Browse	16.70%	24.10%	24.10%

### 2.4 Ethics Approval

This research was approved by the Oklahoma City Zoo and Botanical Garden's Scientific Review Committee (Protocol #2019‐012).

### 2.5 Statistical Analyses

All analyses were conducted in R (R Core Team 2017). To test whether feeding increased, we used a generalized linear mixed‐effects regression model using a beta distribution (‘glmmTMB’ package, Magnusson et al. 2017) for each troop, with gorilla ID, observation session, and hour time block as random effects, condition (year) as the fixed effect, and proportion of intervals spent feeding as the response. To test whether R/R behavior decreased in the family troop, we used a generalized linear model with a Poisson distribution, condition (year) as the fixed effect, and R/R frequency as the response. Due to low occurrence of R/R in the bachelor troop, we used a Fisher's Exact Test with proportion of observation intervals in which R/R occurred as the response variable. We used a zero‐inflated negative binomial regression with a “logit” link to test whether coprophagy behaviors decreased. ID was not included as a random effect due to low occurrence of R/R and coprophagy among the gorillas. We also tested whether behaviors that are not directly related to foraging—such as locomotion, agonistic behaviors, object manipulation, and so forth (Table 2)—changed across the diet conditions by using a generalized linear mixed‐effects regression model using a beta distribution for each troop, with gorilla ID, observation session, and hour time block as random effects, condition (year) as the fixed effect, and proportion of intervals as the response. Assumptions of independence of errors, linearity between transformed response and explanatory variables, and homogeneity of variance were tested and met for these data.

## 3 Results

**Figure 2:**
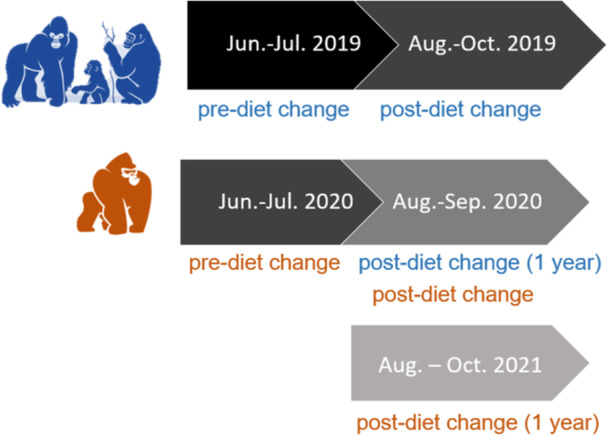
Date ranges of data collection for family (in blue) and bachelor (in orange) troops in this study.

**Figure 3:**
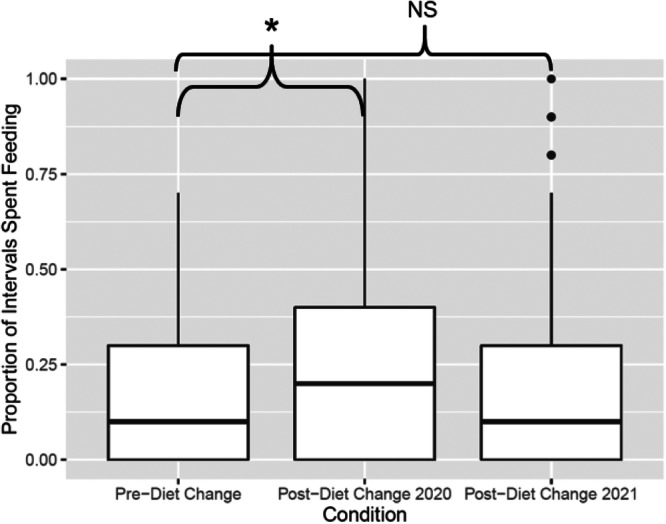
Diet change increased time spent feeding for the bachelor troop, and it remained higher (NS) one year later. Asterisks indicate significant (*p* < 0.001) differences between conditions.

**Figure 4:**
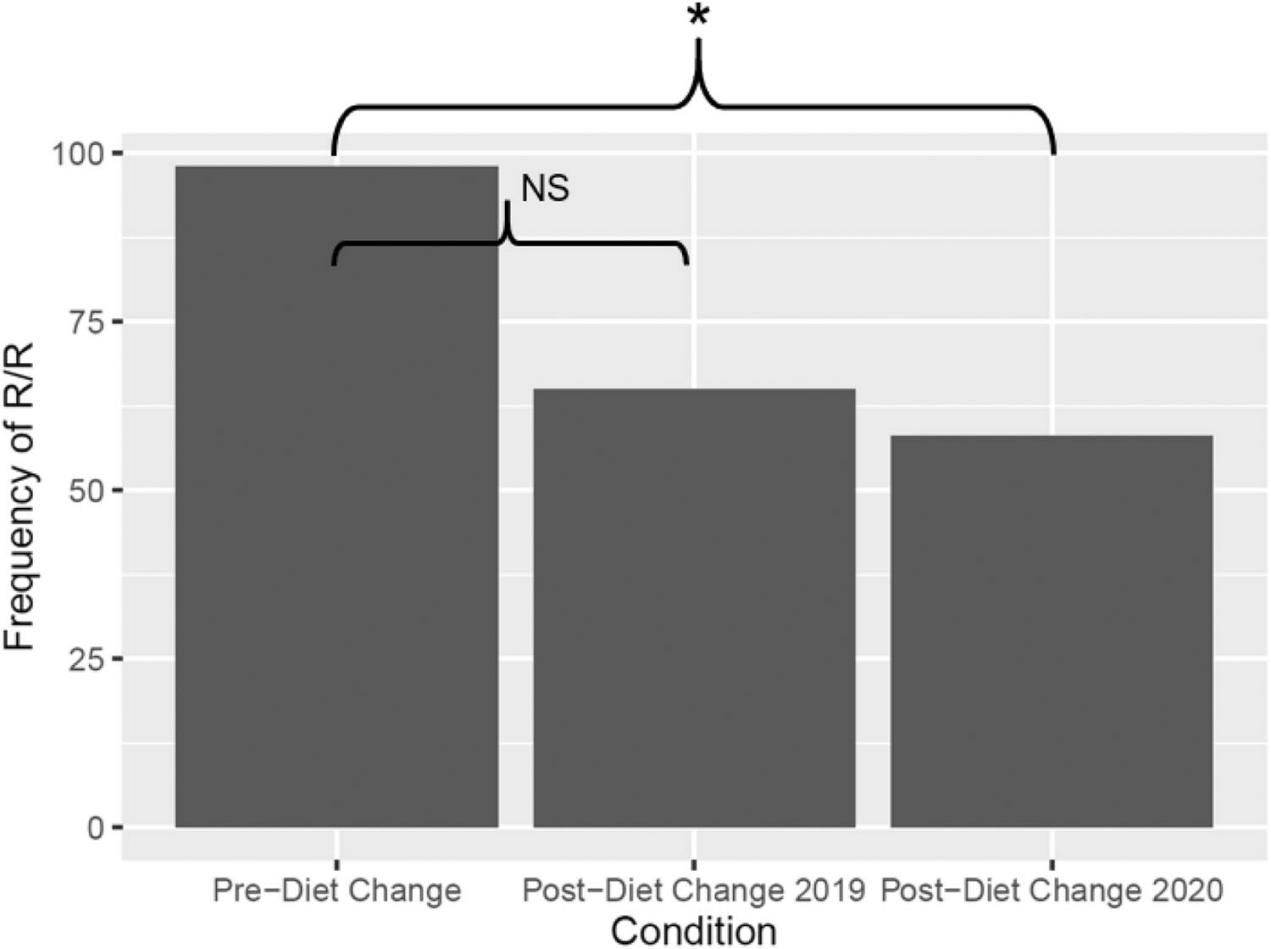
Togo's frequency of R/R decreased after diet change. Asterisk indicates significant (*p* < 0.01) differences between conditions.

**Figure 5:**
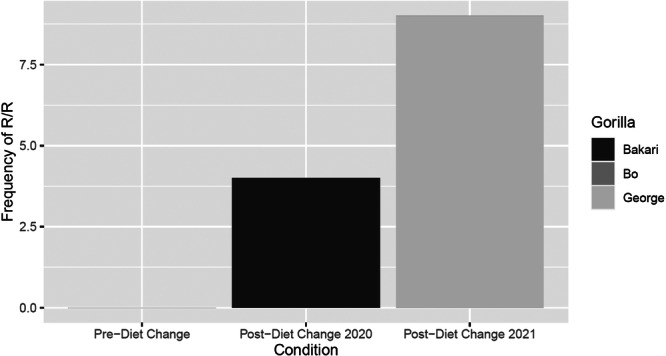
R/R increased after diet change for some individuals in the bachelor troop, Bakari and George.

**Figure 6:**
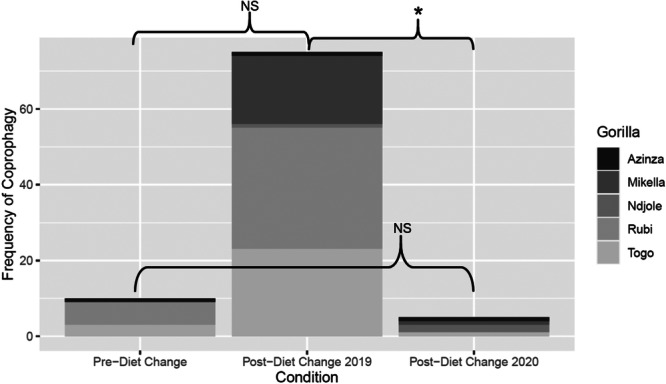
Coprophagy in the family troop increased post‐diet change in 2019 and decreased in 2020. Asterisk indicates significant (*p* < 0.005) differences between conditions.

Time spent feeding significantly increased for the family troop after the diet change in 2019 (*p* < 0.001) compared to pre‐diet change, and it remained significantly higher in 2020 (*p* < 0.001)—after a second diet change—compared to time spent feeding pre‐diet change (Figure 2). Feeding time also significantly increased for the bachelor troop in 2020 (*p* < 0.0001) compared to pre‐diet change, and although feeding remained higher in 2021 than pre‐diet change, this was not statistically significant (*p* = 0.081) (Table 4; Figure 3). In the family troop, we observed only Togo exhibiting R/R behavior (Table 5). Though his R/R was trending down, the behavior did not significantly decrease relative to the baseline period until after the second diet change 1 year later (Table 6; Figure 4). In the bachelor troop, according to a Fisher's Exact Test, there was no significant differences in R/R across the conditions (*p* = 0.079; Figure 5). We observed coprophagy behavior only in the family troop (Table 7). Coprophagy behavior did not significantly change from pre‐diet change to post‐diet change 2019 or to post‐diet change 2020 (Figure 6; Table 8). We also examined whether behaviors not directly related to foraging changed across the diet conditions. For instance, we found that, for the family troop, mobility slightly decreased post‐diet change 2019 but decreased significantly in post‐diet change 2020; there were no significant changes for the bachelor troop (Table 9). Inactivity decreased significantly in the family troop in both post‐diet change conditions, and there was no change in inactivity for the bachelor troop (Table 10). Considering locomotion behavior, there was a slight decrease for the family troop in post‐diet change 2019, and a significant decrease post‐diet change 2020; the bachelor troop decreased locomotion in both post‐diet change conditions (Table 11). Object manipulation was significantly lower post‐diet change 2020 for the family troop and the bachelor troop compared to pre‐diet change (Table 12). There were no significant differences in self‐directed, displace, agonistic, or not visible behaviors among conditions for either troop (Tables 13, 14, 15, 16). The family troop showed significantly more affiliative behaviors post‐diet change 2020 compared to pre‐diet change (Table 17). There were no other significant changes in affiliative behavior (Table 17). The family and bachelor troops showed significantly fewer other behaviors in post‐diet change 2020 compared to prediet change (Table 18). The most common “Other” behavior we observed was urinating/defecating. Raw data can be found in Supplemental Information S2 and S3.

**Table 4:** Output for fixed effects for feeding time. Reference group is pre‐diet change.

Parameter	Estimate	SE	df	*z* value	*p* value
Family troop					
Intercept	0.197	0.042	1149	4.675	< 0.0001
Post‐diet change 2019	0.178	0.043	1149	4.111	< 0.0001
Post‐diet change 2020	0.173	0.043	1149	3.991	< 0.0001
Bachelor troop					
Intercept	0.150	0.051	713	2.960	0.003
Post‐diet change 2020	0.124	0.031	713	4.041	< 0.0001
Post‐diet change 2021	0.053	0.031	713	1.742	0.081

**Table 5:** Average frequency of R/R and standard error (SE) by individual.

Individual	Prediet change mean R/R+/−SE	Postdiet change	Postdiet change
Family troop			
Togo	1.195+/−0.408	0.956+/−0.553	0.725+/−0.285
Bachelor troop			
George	0	0	0.113+/−0.059
Bouendje	0	0	0
Bakari	0	0.050+/−0.039	0

**Table 6:** R/R model output for Togo in the family troop (reference level is pre‐diet change).

Parameter	Estimate	SE	df	*z* value	*p* value
Intercept	0.178	0.101	227	1.765	0.078
Post‐diet change 2019	−0.223	0.160	227	−1.396	0.163
Post‐diet change 2020	−0.500	0.166	227	−3.017	0.003

**Table 7:** Average frequency of coprophagy and standard error (SE) by individual (only observed in the family troop).

Individual	Prediet change mean coprophagy +/− SE	Postdiet change 2019	Postdiet change 2020
Togo	0.037+/−0.027	0.045+/−0.033	0.013+/−0.013
Ndjole	0	0.020+/−0.020	0.025+/−0.018
Mikella	0	0.225+/−0.132	0.013+/−0.013
Rubi	0.073+/−0.045	0.025+/−0.017	0
Azinza	0.012+/−0.012	0.013+/−0.013	0.013+/−0.013

**Table 8:** Family troop's coprophagy model output (reference level is pre‐diet change).

Parameter	Estimate	SE	df	*z* value	*p* value
Intercept	−1.380	1.58	155	−0.873	0.383
Postdiet change 2019	0.533	0.936	155	0.570	0.568
Postdiet change 2020	−3.002	1.648	155	−1.822	0.069
Log(theta)	−1.595	1.755	155	−0.909	0.364

**Table 9:** Model output for mobility behavior (reference condition is pre‐diet change).

Parameter	Estimate	SE	df	*z* value	*p* value
Family troop					
Intercept	0.208	0.036	1146	5.703	< 0.001
Post‐diet change 2019	−0.028	0.017	1146	−1.665	0.096
Post‐diet change 2020	−0.063	0.017	1146	−3.795	< 0.001
Bachelor troop					
Intercept	0.105	0.014	713	7.314	< 0.001
Postdiet change 2020	−0.009	0.013	713	−0.784	0.433
Postdiet change 2021	−0.018	0.013	713	−1.396	0.163

**Table 10:** Model output for inactivity behavior (reference condition is pre‐diet change).

Parameter	Estimate	SE	df	*z* value	*p* value
Family troop					
Intercept	0.430	0.070	1146	6.168	< 0.001
Post‐diet change 2019	−0.133	0.035	1146	−3.790	< 0.001
Post‐diet change 2020	−0.104	0.035	1146	−2.945	0.003
Bachelor troop					
Intercept	0.550	0.038	713	14.578	< 0.001
Post‐diet change 2020	−0.046	0.033	713	−1.392	0.164
Post‐diet change 2021	−0.011	0.033	713	−0.348	0.728

**Table 11:** Model output for locomote behavior (reference condition is pre‐diet change).

Parameter	Estimate	SE	df	*z* value	*p* value
Family troop					
Intercept	0.154	0.026	1146	6.022	< 0.001
Post‐diet change 2019	−0.024	0.014	1146	−1.642	0.101
Post‐diet change 2020	−0.066	0.014	1146	−4.596	< 0.001
Bachelor troop					
Intercept	0.081	0.015	713	5.460	< 0.001
Post‐diet change 2020	−0.022	0.010	713	−2.199	0.028
Post‐diet change 2021	−0.026	0.010	713	−2.666	0.008

**Table 12:** Model output for object‐manipulation behavior (reference condition is pre‐diet change).

Parameter	Estimate	SE	df	z value	*p* value
Family troop					
Intercept	0.028	0.015	1146	1.912	0.056
Post‐diet change 2019	0.005	0.006	1146	0.764	0.445
Post‐diet change 2020	−0.017	0.006	1146	−2.845	0.004
Bachelor troop					
Intercept	0.013	0.004	713	2.856	0.004
Post‐diet change 2020	−0.010	0.004	713	−2.490	0.013
Post‐diet change 2021	−0.003	0.004	713	−0.758	0.448

**Table 13:** Model output for self‐directed behaviors (reference condition is pre‐diet change).

Parameter	Estimate	SE	df	z value	*p* value
Family troop					
Intercept	0.071	0.016	1146	4.418	< 0.001
Post‐diet change 2019	−0.008	0.014	1146	−0.556	0.578
Post‐diet change 2020	−0.009	0.014	1146	−0.647	0.517
Bachelor troop					
Intercept	0.167	0.028	713	5.962	< 0.001
Post‐diet change 2020	−0.034	0.019	713	−1.751	0.080
Post‐diet change 2021	−0.012	0.019	713	−0.631	0.528

**Table 14:** Model output for displace behaviors (reference condition is pre‐diet change). Family troop model contains only gorilla ID as a random intercept due to model convergence issues when Time Block and Session random effects were included. Bachelor model contains gorilla ID and Session because of convergence issues when Time Block was included as a random effect.

Parameter	Estimate	SE	df	z value	*p* value
Family troop					
Intercept	< 0.001	< 0.001	1148	0.000	1.000
Post‐diet change 2019	< 0.001	< 0.001	1148	1.300	0.194
Post‐diet change 2020	< 0.001	< 0.001	1148	1.199	0.230
Bachelor troop					
Intercept	0.003	0.001	714	2.971	0.003
Post‐diet change 2020	−0.003	0.002	714	−1.838	0.066
Post‐diet change 2021	−0.001	0.002	714	−0.788	0.431

**Table 15:** Model output for agonistic behaviors (reference condition is pre‐diet change).

Parameter	Estimate	SE	df	*z* value	*p* value
Family troop					
Intercept	0.004	0.002	1146	2.663	0.008
Post‐diet change 2019	−0.004	0.002	1146	−1.648	0.099
Post‐diet change 2020	−0.004	0.002	1146	−1.751	0.080
Bachelor troop					
Intercept	0.011	0.003	713	3.236	0.001
Postdiet change 2020	−0.007	0.004	713	−1.904	0.057
Post‐diet change 2021	−0.002	0.004	713	−0.562	0.574

**Table 16:** Model output for not visible behaviors (reference condition is pre‐diet change).

Parameter	Estimate	SE	df	*z* value	*p* value
Family Troop					
Intercept	0.022	0.010	1146	2.250	0.024
Post‐diet change 2019	−0.013	0.009	1146	−1.463	0.144
Post‐diet change 2020	0.012	0.009	1146	1.677	0.094
Bachelor troop					
Intercept	< 0.001	0.002	713	0.229	0.819
Post‐diet change 2020	0.004	0.003	713	1.617	0.106
Post‐diet change 2021	0.003	0.003	713	1.132	0.258

**Table 17:** Model output for affiliative behaviors (reference condition is pre‐diet change). Bachelor model contains gorilla ID and Time Block as random effects due to model convergence issues when Session was included as an additional random effect.

Parameter	Estimate	SE	df	*z* value	*p* value
Family troop					
Intercept	0.049	0.031	1146	1.575	0.115
Post‐diet change 2019	0.020	0.013	1146	1.545	0.122
Post‐diet change 2020	0.037	0.013	1146	2.841	0.004
Bachelor troop					
Intercept	< 0.001	< 0.001	715	0.000	1.000
Post‐diet change 2020	< 0.001	< 0.001	715	0.929	0.353
Post‐diet change 2021	< 0.001	< 0.001	715	1.394	0.163

**Table 18:** Model output for other behaviors (reference condition is pre‐diet change).

Parameter	Estimate	SE	df	z value	*p* value
Family troop					
Intercept	0.019	0.008	1146	2.353	0.019
Postdiet change 2019	−0.002	0.004	1146	−0.528	0.597
Post‐diet change 2020	−0.015	0.004	1146	−3.719	< 0.001
Bachelor troop					
Intercept	0.007	0.003	713	2.706	0.007
Post‐diet change 2020	−0.005	0.002	713	−2.476	0.013
Post‐diet change 2021	−0.003	0.002	713	−1.444	0.149

## 4 Discussion

Historically, several different types of primate biscuit were utilized at the Oklahoma City Zoo and Botanical Garden on a rotational basis in the ape diets. Due to changes in purchasing in the animal nutrition center, Marion leaf‐eater biscuit was eliminated from the rotation before the initiation of this study. Anecdotally, R/R behavior decreased in the 30‐year‐old silverback after elimination of this biscuit which was higher in sugar and lower in fiber that the other products fed. We implemented a moderate (i.e., still included some biscuits and fruit) diet change for two troops of gorillas living in human care that were known to exhibit R/R and coprophagy.

We saw a significant increase in time spent feeding in both troops 3 weeks after the diet change and moreover, after 1 year, post‐diet change for the family troop when fruits and vegetables were added back into the diet. Thus, our prediction that the gorillas would spend more time feeding as a result of their new diet (likely due to increased amounts of browse material being offered) was supported. Similar diet change studies (i.e., those seeking to reduce R/R behavior in captive gorillas by presenting more forage/browse) have also documented increases in time spent feeding (e.g., Wiard 1992; Gould and Bres 1986; Fuller et al. 2018; Cabana, Jasmi, and Maguire 2018; Less et al. 2014a). We emphasize the importance of this finding for maintaining positive wellbeing in our troops, especially over such a long time period, given that gorillas in the wild spend a large proportion of their time foraging (Masi, Cipolletta, and Robbins 2009; Magliocca and Gautier‐Hion 2002). For the family troop, this increase in time spent feeding was maintained 1 year after the initial diet change, despite having to alter the diet again in 2020 to include more fruit for positive reinforcement training purposes.

Although R/R and coprophagy increased for some individuals post‐diet change, Togo, who was our stimulus for initiating the diet change, decreased R/R both years, but this was only significant in 2020 (1 year after the diet change) when some fruit was added back into the diet. Moreover, Togo exhibited R/R on fewer days after the diet change. Togo's R/R seemed to be triggered by certain foods he received on those days. In fact, we noticed that small amounts of certain foods provided for training or enrichment (i.e., a single piece of fruit, 0.25 cup of peanuts, or a fruit juice flavored ice treat) was enough to trigger R/R for all individuals that engaged in R/R (i.e., Togo, Bakari, and George). Thus, we urge primate care staff to keep detailed records of which foods—even if infrequently—are provided and whether R/R takes place. Once the behavior has been established, it seems to be easily triggered by preferred foods (Lukas et al. 1999; de Wilde 2024), especially those that are sweet or starchy. We also recommend that future studies examine the type, timing, and amounts of all food items offered to gorillas to better understand how these variables affect R/R. After all, there is individual variation seen in R/R behaviors (e.g., Wiard 1992; Nash et al. 2021).

After a year post‐diet change, coprophagy decreased to levels below pre‐diet change frequencies, although this was not statistically significant. It is possible we will continue to see effects of the diet change as time goes on; researchers have found a similar lagged effect in chimpanzees that did not exhibit any change in R/R in the months after diet change but showed a decrease over a 5‐year period (Wallace et al. 2019). Gorillas in the family troop increased coprophagy behavior 3 weeks after the diet change. This type of initial spike in coprophagy has been found elsewhere (Less et al. 2014a).

It is also possible that changes in access to conspecifics (vs. diet) contributed to behavioral changes in the family troop. When the surrogate mother‐son pair were separated from the rest of the family troop in 2019, we observed that the troop often chose to stay in their indoor enclosure during this time, possibly because they could see and hear their conspecifics from this location. Separation from troop members may have been a source of stress, and stress‐related behaviors have been shown to be associated with R/R in other ape species (Miller and Tobey 2012). However, we did not observe any other stress‐related behaviors (e.g., hair‐plucking, ear cupping/covering) in the troop in 2019. We recommend that future studies continue to monitor behavior, ideally for several years, after the initial diet change to more accurately capture effects of diet outside of confounding factors such as housing changes.

Notably, we retained some biscuits and fruits in our revised diet, which makes this diet potentially less costly and more manageable compared to previously proposed diet changes, such as those which eliminated all biscuits (e.g., AZA Gorilla Species Survival Plan 2017; Less et al. 2014a, 2014b; Lukas 1999). Our low sample size restricts the generalization of these results, but given that many independent studies have found similar results, it is clearly beneficial to increase fiber and reduce starch for gorillas in human care. Overall, we recommend lower‐starch, higher‐fiber diets—even moderately higher‐fiber diets—for other institutions seeking to increase time spent feeding and reduce R/R in their great apes.

## Supporting information

Video S1. Video clip of R/R behavior in male gorilla at Oklahoma City Zoo and Botanical Garden.

Supplemental Information 1. Original and revised diet items, amounts, and macronutrient breakdowns.

Supplemental Information 2. Raw data from bachelor troop.

Supplemental Information 3. Raw data from family troop.

## Data Availability

All relevant data is contained within the article: The original contributions presented in the study are included in the supplementary material. Further inquiries can be directed to the corresponding author.
